# Greater Cognitive Decline with Aging among Elders with High Serum Concentrations of Organochlorine Pesticides

**DOI:** 10.1371/journal.pone.0130623

**Published:** 2015-06-24

**Authors:** Se-A Kim, Yu-Mi Lee, Ho-Won Lee, David R Jacobs, Duk-Hee Lee

**Affiliations:** 1 Department of Biomedical Science, Kyungpook National University, Daegu, Korea; 2 BK21 Plus KNU Biomedical Convergence Program, Department of Biomedical Science, Kyungpook National University, Daegu, Korea; 3 Department of Preventive Medicine, School of Medicine, Kyungpook National University, Daegu, Korea; 4 Department of Neurology, School of Medicine, Kyungpook National University, Daegu, Korea; 5 Brain Science & Engineering Institute, Kyungpook National University, Daegu, Korea; 6 Division of Epidemiology, School of Public Health, University of Minnesota, Minneapolis, Minnesota, United States of America; Institute for Health & the Environment, UNITED STATES

## Abstract

Although cognitive decline is very common in elders, age-related cognitive decline substantially differs among elders and the determinants of the differences in age-related cognitive decline are unclear. We investigated our hypothesis that the association between age and cognition was stronger in those with higher serum concentrations of organochlorine (OC) pesticides, common persistent and strongly lipophilic neurotoxic chemicals. Participants were 644 elders aged 60-85, participating in the National Health and Nutrition Examination Survey 1999-2002. Six OC pesticides (p,p'-dichlorodiphenyltrichloroethane (DDT), p,p'-dichlorodipenyldichloroethylene (DDE), β-hexachlorocyclohexane, trans-nonachlor, oxychlordane, and heptachlor epoxide) were evaluated. “Lower cognitive function” was defined as having a low Digit-Symbol Substitution Test (DSST) score (<25^th^ percentile of DSST score, cutpoint 28 symbols substituted). Higher levels of β-hexachlorocyclohexane, trans-nonachlor, oxychlordane, and heptachlor epoxide modified the associations between age and lower cognitive function (P_interaction_<0.01, 0.03, <0.01, and 0.02, respectively). Elders in the 3^rd^ tertile of these chemicals demonstrated a greater risk of lower cognitive function with aging, compared to those in the combined 1^st^ and 2^nd^ tertiles. Among those with highest OC pesticides (3^rd^ tertile), the odds ratio for the risk of lower cognitive function was about 6 to 11 for the highest quintile of age (80-85 years) vs. the first quintile of age (60-63 years), while the association between age and lower cognitive function became flatter in those with lower OC pesticides (combined 1^st^ and 2^nd^ tertiles). Both DDT and DDE showed no interaction, with lower DSST scores for higher age irrespective of serum concentrations of DDT or DDE. Even though DSST score measures only one aspect of cognition, several OC pesticides modified aging-related prevalence of low cognitive score, a finding which should be evaluated in prospective studies.

## Introduction

Several recent studies found that background exposure to organochlorine (OC) pesticides in general populations was strongly associated with the risk of low cognition or dementia in elders [1–3]. As known neurotoxins [4], OC were used extensively from the 1940s through the 1960s in agriculture and mosquito control, but banned in most developed countries after the 1970s due to possible harms to environment, wild animals and humans [5]. However, their unique characteristics, including strong lipophilicity, poor biodegradation, and biomagnification in the food chain, make these chemicals an ongoing concern to human health in the 21th century [6].

Despite being banned several decades ago, people are presently continuously exposed to OC pesticides through two main exposure sources [6]. First, OC pesticides stored in adipose tissue during earlier high exposure periods continuously leach out of the fat compartment, reaching critical organs like the brain. Second, because of persistence of OC pesticides in biological tissues, the consumption of OC pesticide contaminated food like fatty animal food has become an important external exposure source. Importantly, elders today are the first generation with a whole life-time exposure to OC pesticides including the highest exposure period in human history. As neurotoxic, fat-soluble, pesticides which are not easily metabolized and which cross the blood-brain barrier, it is plausible that there is acceleration of cognitive loss with aging in those with greater exposure to OC pesticides.

Cognitive decline is very common in the general elderly population and increases with age [7–9]. However, age-related cognitive decline substantially differs among elders and the determinants of the differences in age-related cognitive decline are unclear [10]. Although individual differences in cognitive aging were related to many risk factors such as genetics, general health and medical disorders, diet, and lifestyle in previous studies, many of these effect sizes are small and some are poorly replicated [10].

Therefore, we investigated whether chronic exposure to OC pesticides can modify aging-related cognitive decline among U.S. elders who did not have overt dementia. In our previous study based on the same dataset, we reported strong associations between some OC pesticides and cognitive impairment [1]. In this study, we further hypothesized that aging-related cognitive decline would be greater among elders with higher exposure to OC pesticides than among those with lower OC pesticide exposure.

## Materials and Methods

### Participants

The continuous National Health and Nutrition Examination Survey (NHANES) conducted annually since 1999 by the Centers for Disease Control and Prevention (CDC), is an ongoing survey designed to measure the health and nutritional status of the civilian noninstitutionalized U.S. population. Serum organochlorine pesticides were measured in the subsample of the NHANES 1999–2004 while cognitive function was measured in elders aged 60~85 of the NHANES 1999–2002. Thus, the final sample size was 644 who had information on both cognitive function and organochlorine pesticides. The study protocol was reviewed and approved by the institutional review board of the Centers for Disease Control in the U.S. Also, informed written consent was obtained from all subjects before they took part in the study.

### Cognitive performance

Cognitive function was assessed using a single test, the Digit Symbol Substitution Test (DSST), during the NHANES household interview. The DSST is a component of the Wechsler Adult Intelligence Test. It is a test of visuospatial and motor speed-of-processing, has a considerable executive function component and is frequently used as a sensitive measure of frontal lobe executive function [11]. The task consisted of drawing unfamiliar symbols under the corresponding number, resulting in a score indicating the number of correct symbols drawn within a period of 120 seconds with a maximum score of 133. Sample items were provided for initial practice; participants who were unable to complete the sample items for any reason did not continue with the remainder of the test.

### Measurement

Venous blood samples were collected and shipped weekly at -20°C. Organochlorine pesticides were all measured as individual chemicals by high-resolution gas chromatography/high-resolution mass spectrometry using isotope dilution for quantification. We selected 6 organochlorine pesticides (p,p’- dichlorodiphenyltrichloroethane (DDT), p,p’- dichlorodipenyldichloroethylene (DDE), β-hexachlorocyclohexane, trans-nonachlor, oxychlordane, and heptachlor epoxide) which were associated with DSST score in a previous study [1]. For lipid-standardized concentrations, each organochlorine concentration was divided by the total lipid value (Total lipids (mg/dl) = 2.27 x total cholesterol+triglycerides+62.3). Results of lipid-standardized concentrations were similar to those of wet concentrations adjusted for lipids in statistical models [1]. We presented results based on lipid-standardized concentrations.

### Statistical Analysis

In this study, we used both continuous DSST score and dichotomous DSST score < 25% percentile as study outcomes. The mean of the continuous measure captured the general population tendencies in DSST score, while the dichotomy was more appropriate for investigating associations that might relate to more serious cognitive impairment.

Thus, we evaluated if serum concentrations of 6 OC pesticides (tertiles) modified associations between age (quintiles) and continuous DSST score using general linear models, then further evaluated the dichotomous outcome of cognitive score < 25% percentile using logistic regressions. For the latter analyses, we combined the 1^st^ and 2^nd^ tertiles of OC pesticides to increase statistical stability after noting that the patterns of associations were similar between these two tertiles (data not shown). Adjusting covariates were gender, race-ethnicity (non-Hispanic white, non-Hispanic black, Mexican American, multiracial, or others), education (less than 9^th^ grade, 9^th^-11^th^ grade, and high school graduates), poverty income ratio (the ratio of self-reported family income to the family’s appropriate threshold value, continuous), smoking status (current, ex, and never), body mass index (continuous), physician-diagnosed heart disease, physician-diagnosed hypertension, and physician-diagnosed diabetes.

Estimates of main results were calculated accounting for NHANES stratification and clustering [12], adjusting for age, gender, and race-ethnicity instead of using sample weights; this adjustment has been regarded as a good compromise between efficiency and bias [12, 13]. We used SAS statistical software (version 9.3; SAS Institute, Cary, NC).

## Results

Characteristics of study subjects by quintiles of age are presented in Table 1. Proportions of white race and prevalence of physician-diagnosed heart disease and hypertension were significantly higher with age while those of current smoker and obesity were lower. The DSST score was also significantly lower with aging and the difference of DSST score between the lowest and highest age quintiles was about 16 symbols correctly substituted.

**Table 1:** Characteristics of study subjects by quintiles of age (n = 644).

	Quintiles of age (years)					
Characteristics	Q1 (60–63)	Q2 (64–67)	Q3 (68–72)	Q4 (73–79)	Q5 (80–85)	P_trend_
	(n = 134)	(n = 121)	(n = 124)	(n = 128)	(n = 137)	
Mean ± standard deviation						
Age, years	61.3±1.1	65.5±1.1	69.9±1.3	75.6±2.0	82.7±2.0	<0.01
Poverty income ratio	2.6±1.5	2.3±1.2	2.1±1.3	2.4±1.4	2.4±1.3	0.07
Body mass index, kg/m^2^	30.1±6.4	29.9±5.9	28.5±5.1	27.6±4.4	25.9±3.9	<0.01
Score of Digit Symbol Substitution Test	48.9±20.6	43.0±17.3	40.8±18.3	39.0±16.4	32.8±15.1	<0.01
Number of subjects (%)						
Men	54 (40.3%)	60 (49.6%)	61 (49.2%)	72 (56.3%)	56 (40.9%)	0.05
White	58 (43.3%)	56 (46.3%)	61 (49.2%)	90 (70.3%)	118 (86.1%)	<0.01
High school graduate	48 (35.8%)	42 (34.7%)	33 (26.6%)	42 (32.8%)	54 (39.4%)	0.23
Current smoker	30 (22.4%)	12 (9.9%)	14 (11.3%)	6 (4.7%)	9 (6.6%)	<0.01
Body mass index ≥ 30 kg/m^2^	50 (37.3%)	49 (40.5%)	44 (35.5%)	38 (29.7%)	12 (8.8%)	<0.01
Physician-diagnosed heart disease	12 (9.0%)	18 (14.9%)	23 (18.6%)	28 (21.9%)	41 (29.9%)	<0.01
Physician-diagnosed diabetes	37 (27.6%)	31 (26.6%)	38 (30.7%)	23 (18.0%)	26 (19.0%)	0.07
Physician-diagnosed hypertension	83 (61.9%)	72 (59.5%)	90 (72.6%)	87 (68.0%)	105 (76.6%)	0.02

Table 2 shows associations between age and mean DSST score stratified by tertiles of serum concentrations of each OC pesticides. Even though all 3 strata showed significant associations between age and mean DSST score, p values for interaction between age and OC pesticides were significant for β-hexachlorocyclohexane, trans-nonachlor, and oxychlordane. For these chemicals, the negative associations between age and mean DSST score generally strengthened (accelerated aging) at higher serum concentrations of these OC pesticides (P for interaction = 0.04 for β-hexachlorocyclohexane, 0.05 for trans-nonachlor, and 0.02 for oxychlordane). Looked at alternatively, mean DSST score was lower in those with higher OC pesticides only in the older age group for these three chemicals, with a similar tendency for heptachlor epoxide.

**Table 2:** Adjusted* means of the Digit Symbol Substitution Test score by quintiles of age according to tertiles of serum concentrations of organochlorine pesticides.

	Tertile	Quintiles of age (years)							
	(median concentration,	Q1	Q2	Q3	Q4	Q5	Q5-Q1	P_trend_	P_interaction_
	ng/g lipid)	(60–63)	(64–67)	(68–72)	(73–79)	(80–85)			
		(n = 134)	(n = 121)	(n = 124)	(n = 128)	(n = 137)			
P,p’-DDT	T1 (5.7)	52.3	46.0	51.2	41.4	35.2	17.1	<0.01	0.22
	T2 (9.4)	50.4	45.0	43.0	41.3	33.7	16.7	<0.01	
	T3 (25.6)	36.4	38.9	35.8	36.4	26.5	9.9	<0.01	
P,p’-DDE	T1 (324.5)	51.0	46.9	46.3	40.5	35.5	15.5	<0.01	0.64
	T2 (940.5)	48.8	40.9	45.3	43.0	31.4	17.4	<0.01	
	T3 (2200.0)	40.5	41.9	37.9	35.8	28.8	11.7	<0.01	
β-hexachlorocyclohexane	T1 (12.8)	48.6	43.4	46.1	41.2	36.7	11.9	<0.01	0.04
	T2 (28.5)	46.9	46.2	43.2	41.6	31.6	15.3	<0.01	
	T3 (73.4)	43.3	40.3	39.4	35.9	29.6	13.7	<0.01	
Trans-nonachlor	T1 (25.9)	47.6	43.1	45.3	41.4	35.9	11.7	0.01	0.05
	T2 (48.2)	45.1	46.6	44.8	41.8	33.5	11.6	<0.01	
	T3 (88.9)	46.4	40.5	38.4	34.8	29.4	17.0	<0.01	
Oxychlordane	T1 (17.9)	49.4	41.8	45.0	39.1	41.3	8.1	<0.01	0.02
	T2 (31.3)	47.1	48.3	45.1	43.7	31.6	15.5	<0.01	
	T3 (54.7)	42.5	39.2	39.0	35.4	28.8	13.7	<0.01	
Heptachlor epoxide	T1 (5.1)	48.1	42.1	42.7	38.4	39.1	9.0	<0.01	0.20
	T2 (10.7)	44.7	48.5	46.5	45.0	34.6	10.1	<0.01	
	T3 (23.0)	46.2	38.7	39.3	35.7	28.8	17.4	<0.01	

* Adjusted for age, sex, race-ethnicity, education, poverty income ratio, cigarette smoking, body mass index, physician-diagnosed heart disease, physician-diagnosed hypertension, and physician-diagnosed diabetes

The nonsignificant p values for interaction for p,p’-DDT and p,p’-DDE reflected consistently lower mean DSST score at higher ages and, independently, at higher p,p’-DDT and p,p’-DDE concentrations. Thus, compared to other OC pesticides, higher serum concentrations of p,p’-DDT and p,p’-DDE were strongly associated with lower mean DSST scores even among elders aged 60 to 63.

Patterns of interaction were even clearer for the risk of having an absolutely low cognitive score, defined as DSST score < 25% percentile (28 symbols correctly substituted) as the outcome than for the mean DSST score (Table 3). Except for p,p’-DDT, and p,p’-DDE, all 4 other OC pesticides showed statistically significant interactions. Among elders in the 1^st^ or 2^nd^ tertiles of β-hexachlorocyclohexane, trans-nonachlor, oxychlordane, and heptachlor epoxide, there was no clear association between age and the risk of low DSST score. However, when elders had serum concentrations of these OC pesticides in the 3^rd^ tertile, there was greater accelerated aging in progressively higher prevalence of low DSST score; adjusted ORs in the highest quintile of age (80–85 years old) vs the lowest quintile of age (60–63 years old) were 11.4 (95% confidence interval (CI): 2.7–48.0) for β-hexachlorocyclohexane, 6.7 (1.7–26.4) for trans-nonachlor, 10.1 (2.4–41.9) for oxychlordane, and 6.1 (1.7–21.6) for heptachlor epoxide. These age patterns were closer to flat for these same chemicals in people with lower OC pesticide concentrations. On the other hand, for p,p’-DDT or p,p’-DDE, increasing prevalence of lowest quartile DSST scores was seen across ages, both in elders within the 1^st^ or 2^nd^ OC pesticide tertiles and within the 3^rd^ OC pesticide tertile, similar to the results for mean DSST score, Looked at alternatively, prevalence of low DSST score got higher as either p,p’-DDT or p,p’-DDE got higher, within most age groups.

**Table 3:** Adjusted* odds ratios (ORs) and 95% confidence intervals (CIs) between age and the risk of low Digit Symbol Substitution Test score (<25^th^ percentile of all study subjects, 28 symbols correctly substituted) according to serum concentrations of organochlorine pesticides.

			Quintiles of age (years)						
			Q1 (60–63)	Q2 (64–67)	Q3 (68–72)	Q4 (73–79)	Q5 (80–85)	P_trend_	P_interaction_
			(n = 134)	(n = 121)	(n = 124)	(n = 128)	(n = 137)		
P,p’-DDT	T1+T2	Cases/Subjects	12/94	9/75	15/84	18/87	32/91		
		Prevalence (%)	12.8	12	17.9	20.7	35.2		
		Adjusted OR	Reference	0.6 (0.2–1.6)	0.8 (0.3–2.0)	1.6 (0.6-.4.0)	4.2 (1.7–10.9)	<0.01	
	T3	Cases/Subjects	14/40	16/46	17/40	15/41	20/46		
		Prevalence (%)	35.0	34.8	42.5	36.6	43.5		
		Adjusted OR	Reference	0.8 (0.4–1.6)	0.8 (0.4–1.7)	1.2 (0.6–2.4)	3.1 (0.9–10.5)	0.25	0.37
P,p’-DDE	T1+T2	Cases/Subjects	12/85	13/77	13/84	19/89	35/93		
		Prevalence (%)	14.1	16.9	15.5	21.3	37.6		
		Adjusted OR	Reference	1.0 (0.4–2.8)	0.8 (0.3–2.1)	1.6 (0.6–4.1)	4.7 (1.8–12.5)	<0.01	0.73
	T3	Cases/Subjects	14/49	12/44	19/40	14/39	17/44		
		Prevalence (%)	28.6	27.3	47.5	35.9	38.6		
		Adjusted OR	Reference	0.8 (0.3–2.7)	0.8 (0.3–2.7)	0.8 (0.3–2.6)	2.7 (0.8–9.1)	0.20	
β-hexachlorocyclohexane	T1+T2	Cases/Subjects	21/95	15/79	18/85	20/94	23/76		
		Prevalence (%)	22.1	19.0	21.2	21.3	30.3		
		Adjusted OR	Reference	0.5 (0.2–1.1)	0.4 (0.2–1.1)	0.7 (0.3-.1.6)	1.6 (0.7–3.9)	0.30	<0.01
	T3	Cases/Subjects	5/39	10/42	14/39	13/34	29/61		
		Prevalence (%)	12.8	23.8	35.9	38.2	47.5		
		Adjusted OR	Reference	2.6 (0.7–10.5)	2.7 (0.7–10.8)	5.1 (1.2–21.4)	11.4 (2.7–48.0)	<0.01	
Trans-nonachlor	T1+T2	Cases/Subjects	19/99	16/76	20/90	18/86	22/78		
		Prevalence (%)	19.2	21.1	22.2	20.9	28.2		
		Adjusted OR	Reference	0.7 (0.3–1.7)	0.8 (0.4–1.9)	1.2 (0.5–2.9)	2.1 (0.9–5.1)	0.06	0.03
	T3	Cases/Subjects	7/35	9/45	12/34	15/42	30/59		
		Prevalence (%)	20.0	20.0	35.3	35.7	50.8		
		Adjusted OR	Reference	1.4 (0.4–5.3)	0.9 (0.2–3.7)	1.5 (0.4–5.6)	6.7 (1.7–26.4)	<0.01	
Oxychlordane	T1+T2	Cases/Subjects	22/102	17/86	21/94	18/81	18/66		
		Prevalence (%)	21.6	19.8	22.3	22.2	27.3		
		Adjusted OR	Reference	0.6 (0.2–1.3)	0.6 (0.3–1.4)	1.0 (0.4–2.4)	1.7 (0.7–4.3)	0.20	<0.01
	T3	Cases/Subjects	4/32	8/35	11/30	15/47	34/71		
		Prevalence (%)	12.5	22.9	36.7	31.9	47.9		
		Adjusted OR	Reference	3.0 (0.6–14.1)	2.5 (0.6–11.3)	2.2 (0.5–9.2)	10.1 (2.4–41.9)	<0.01	
Heptachlor epoxide	T1+T2	Cases/Subjects	18/95	16/76	20/91	18/87	23/82		
		Prevalence (%)	18.9	21.1	22.0	20.7	28.0		
		Adjusted OR	Reference	0.7 (0.3–1.8)	0.7 (0.3–1.6)	0.8 (0.3–1.9)	1.9 (0.8–4.8)	0.26	0.02
	T3	Cases/Subjects	8/39	9/45	12/33	15/41	29/55		
		Prevalence (%)	20.5	20.0	36.4	36.6	52.7		
		Adjusted OR	Reference	0.9 (0.3–3.1)	1.2 (0.3–4.5)	2.2 (0.7–7.5)	6.1 (1.7–21.6)	<0.01	

*Adjusted for age, sex, race-ethnicity, education, poverty income ratio, cigarette smoking, body mass index, physician-diagnosed heart disease, physician-diagnosed hypertension, and physician-diagnosed diabetes

**Fig 1:**
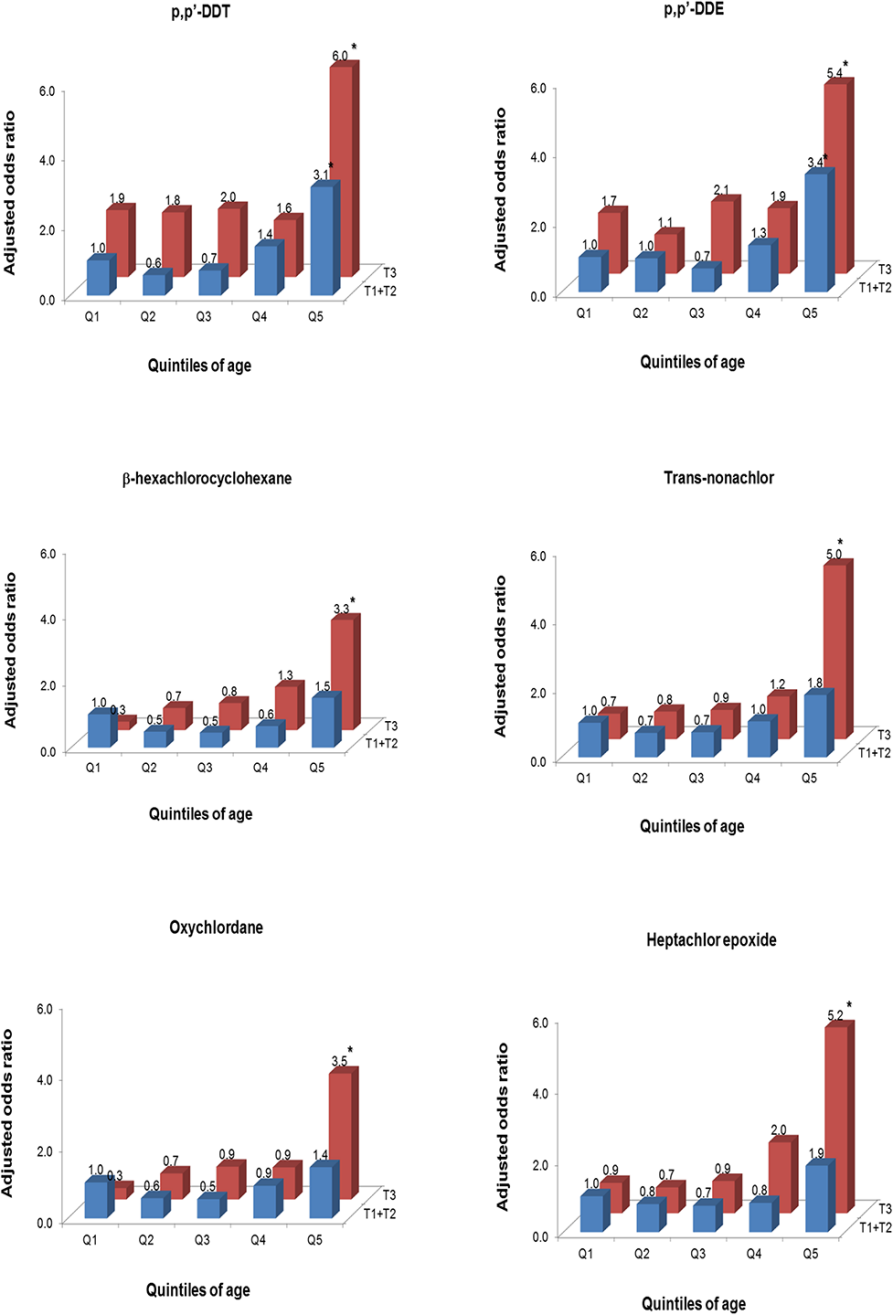
Associations of age and 6 organochlorine (OC) pesticides on the risk of low cognitive score (<25th percentile of study subjects, 28 of 133 symbols correctly substituted). Odds ratios were estimated using logistic regression with a common reference group of elderly in the 1^st^ quintile of age and the combined 1st plus 2^nd^ tertile of each OC pesticide, adjusted for age, sex, race-ethnicity, education, poverty income ratio, cigarette smoking, body mass index, physician-diagnosed heart disease, physician-diagnosed hypertension, and physician-diagnosed diabetes. Statistically significant odds ratios were marked with *. Q1, first quintile (60–63 years); Q2, second quintile (64–67 years); Q3, third quintile (68–72 years); Q4, forth quintile (73–79 years); Q5, fifth quintile (80–85 years); T1+T1, first and second tertiles; T3, third tertile

Fig 1 shows adjusted ORs when elders in the 1st quintile of age (60–63 years old) who were also in the 1st or 2nd tertiles of each OC pesticide were used as a common reference group. The strongest risk of low DSST score was observed in the highest quintile of age (80–85 years old) with the highest OC pesticides. For p,p’-DDT and p,p’-DDE, when elders had serum concentrations of these compounds belonging to the 3^rd^ tertile, the risk of low cognition even among early elders in the 1^st^ quintile of age was somewhat higher. Similar to the results for DSST score, this fact seemed to contribute to the weak associations between age and low cognitive score among elders with the 3^rd^ tertile of these chemicals in the stratified analyses of Table 3.

## Discussion

This study demonstrated that β-hexachlorocyclohexane, trans-nonachlor, oxychlordane, and heptachlor epoxide modified the well-known association between age and cognitive function in U.S. elders without overt dementia. The inverse associations between age and DSST score accelerated as serum concentrations of these OC pesticides increased. These patterns were more clearly observed with the risk of low cognition defined as <25^th^ percentile of DSST score, than for the general population tendencies reflected in the mean DSST score For example, we observed accelerated aging in prevalence of low DSST score when elders had serum concentrations of these chemicals in the 3^rd^ OC pesticide tertile. Although inverse age associations were found for the mean DSST score among elders with low levels of these OC pesticides, age associations with prevalence of low DSST score were relatively flat in people with lower OC pesticide concentrations.

Researchers have suggested some intrinsic and extrinsic factors which can modulate cognitive decline with aging, such as gender, education, apoplipoprotein E, physical activity, or food intake [14–16]. However, we found no studies of the role of OC pesticides in this context yet. Furthermore, compared to other modifying factors, the role of OC pesticides in accelerated cognitive aging has the interesting feature that there was only a weak age association with prevalent low DSST score among subjects with low levels of OC pesticides. Although there is no standard linking a particular DSST score to cognitive impairment, this finding is suggestive that having low OC pesticide exposure delays passage into a practically impaired state.

In this study, we used two outcomes, continuous DSST score and dichotomous low cognition defined as <25^th^ percentile of DSST score. As elders who were able to complete the sample items provided DSST score, the results focusing on DSST score may be more relevant to non-pathological normal age-related cognitive decline. Thus, in the case of findings about mean DSST score, we can interpret that OC pesticides like β-hexachlorocyclohexane, trans-nonachlor, oxychlordane, and heptachlor epoxide can be contributors to individual differences in normal cognitive aging. However, DSST score <25th percentile represents a more serious stage of cognitive decline in which there could be clinical implications. Even though we evaluated only one cognitive function among various domains of cognitive function, various test scores used for neuropsychological assessment are often correlated because common cognitive components are involved in different tests [17]. Also, a low DSST score in older adults who are functioning well enough to complete the DSST test could predict mortality and disability, independent of other risk factors, including measures of brain integrity [18]. In this sense, little association between age and the risk of DSST score <25th percentile among elders with low serum concentrations of these OC pesticides suggests low exposure to these pesticides may delay a wide variety of adverse outcomes mental and physical health in the elderly.

In fact, in our previous study based on the same dataset, we evaluated the main association between OC pesticides and cognitive function after adjusting covariates including age [1]. In that study, p,p’-DDT, p,p’-DDE, and β-hexachlorocyclohexane were associated with both mean DSST score and the risk of low DSST score. However, trans-nonachlor, oxychlordane, and heptachlor epoxide were associated only with mean DSST score, but not the risk of low DSST score. When we evaluated the role of OC pesticides in accelerated cognitive aging in the current study, the compounds not associated with the risk of low cognition in the previous study showed statistically significant interactions with age. Thus, if these findings were interpreted from the viewpoint of OC pesticides, the associations between these compounds and low cognition became prominent only at later elderly ages, but not earlier in their lives. This helps to explain why trans-nonachlor, oxychlordane, and heptachlor epoxide were not related to the risk of low cognition in the previous study [1].

On the other hand, DDT-related compounds did not show statistically significant interactions with age. DDT-related compounds were found across all ages to be associated with lower mean DSST score and with prevalence of lowest quartile DSST score. Thus, this finding can be interpreted to suggest the importance of DDT on cognitive function irrespective of age. This finding is consistent with our previous study, in which p,p’-DDT showed the strongest association among 6 OC pesticides. For example, elders with p,p’-DDT in the highest 5th percentile showed 6.5 times higher risk of low cognitive score.

This study has several limitations. First, the cross-sectional study design precludes the establishment of temporal relationships even though the interaction between age and OC pesticides on the risk of low cognitive function may be difficult to explain by reverse causality. Second, only DSST was used to evaluate cognitive function. Cognition is generally assessed through a battery of psychometric tests repeatedly administered to the subjects. Collecting several cognitive tests may be useful because this allows exploration of the various cognitive domains. Third, the outcome used in this study was not definite dementia. Even though cognitive function in older people is reportedly a predictor of dementia [17], future studies with clinically-relevant outcomes are required.

## Conclusions

As the population ages, burdens and costs to individuals and society arising from late-life cognitive decline are alarming. However, the present study demonstrated that the association between age and cognitive impairment could be modified by the levels of certain OC pesticides. The role of OC pesticides in aging-related cognitive decline should be evaluated in prospective human studies. Also, molecular mechanisms on possible neurotoxicity of low dose chronic exposure need to be investigated in experimental studies.
